# A genetic titration of membrane composition in *Caenorhabditis elegans* reveals its importance for multiple cellular and physiological traits

**DOI:** 10.1093/genetics/iyab093

**Published:** 2021-06-14

**Authors:** Ranjan Devkota, Delaney Kaper, Rakesh Bodhicharla, Marcus Henricsson, Jan Borén, Marc Pilon

**Affiliations:** 1 Department of Chemistry and Molecular Biology, University of Gothenburg, Gothenburg S-405 30, Sweden; 2 Department of Molecular and Clinical Medicine/Wallenberg Laboratory, Institute of Medicine, University of Gothenburg, Gothenburg S-405 30, Sweden

**Keywords:** phospholipid, cell membrane, membrane fluidity, autophagy, lifespan, lipid peroxidation, AdipoR, germline, vitellogenin, permeability, lipidomics

## Abstract

Communicating editor: B. Grant

The composition and biophysical properties of cellular membranes must be tightly regulated to maintain the proper functions of myriad processes within cells. To better understand the importance of membrane homeostasis, we assembled a panel of five *Caenorhabditis elegans* strains that show a wide span of membrane composition and properties, ranging from excessively rich in saturated fatty acids (SFAs) and rigid to excessively rich in polyunsaturated fatty acids (PUFAs) and fluid. The genotypes of the five strain are, from most rigid to most fluid: *paqr-1(tm3262); paqr-2(tm3410), paqr-2(tm3410)*, N2 (wild-type), *mdt-15(et14); nhr-49(et8)*, and *mdt-15(et14); nhr-49(et8); acs-13(et54)*. We confirmed the excess SFA/rigidity-to-excess PUFA/fluidity gradient using the methods of fluorescence recovery after photobleaching (FRAP) and lipidomics analysis. The five strains were then studied for a variety of cellular and physiological traits and found to exhibit defects in: permeability, lipid peroxidation, growth at different temperatures, tolerance to SFA-rich diets, lifespan, brood size, vitellogenin trafficking, oogenesis, and autophagy during starvation. The excessively rigid strains often exhibited defects in opposite directions compared to the excessively fluid strains. We conclude that deviation from wild-type membrane homeostasis is pleiotropically deleterious for numerous cellular/physiological traits. The strains introduced here should prove useful to further study the cellular and physiological consequences of impaired membrane homeostasis.

## Introduction 

Cell membrane homeostasis ensures near-optimal membrane composition and properties among the distinct membranous structures of the cell. Generally, there is an increasing gradient of rigidity/saturation from the nuclear outer membrane all the way to the plasma membrane, which is the most rigid and saturated fatty acid (SFA)-rich membrane (Antonny *et al.* 2015). Although most fatty acids (FAs) are obtained from the diet, the FA composition of membrane phospholipids is remarkably resistant to changes in the dietary FA composition, which is particularly true of SFAs. For example, a thorough dietary intervention study in rats showed that the membrane SFA content remained stable even when the dietary SFA content varied from ∼10 to ∼90% (Abbott *et al.* 2012). In addition, when membrane composition does change in response to polyunsaturated fatty acid (PUFA)-rich diets, there are usually compensatory changes that maintain membrane properties, such as membrane packing (Levental *et al.* 2020). Such robust homeostasis of membrane composition suggests that this is crucial for cellular/physiological health and that effective mechanisms must exist that guard membrane composition/properties against detrimental fluctuations.

Elegant studies in recent years revealed that proteins acting in different cellular compartments contribute to membrane homeostasis (de Mendoza and Pilon 2019). From the nucleus outwards, they include: PCYT1A that senses membrane curvature/packing defects in the nuclear membrane and regulates compensatory phosphatidylcholine (PC) synthesis (Haider *et al.* 2018), IRE1 that monitors ER membrane thickness and thus indirectly its degree of saturation (Halbleib *et al.* 2017), and the AdipoR proteins in the plasma membrane that are activated by decreased fluidity and respond by promoting increased desaturation in phospholipids (Devkota *et al.* 2017; Ruiz *et al.* 2019b). In addition, the SREBP1 and SREBP2 transcription factors are regulated by membrane defects and depletion of cholesterol or unsaturated fatty acids (UFAs) and act as an additional regulatory layer for membrane homeostasis (Hannah *et al.* 2001; Walker *et al.* 2011; Shimano and Sato 2017).

That multiple molecular sensors and regulators act to maintain membrane homeostasis suggests that it is paramount to cellular health. Although few systematic studies of the consequences of membrane homeostasis impairment have been done, separate reports from various experimental systems indicate that membrane composition/property defects have important consequences. Insulin receptor properties and trafficking of the GLUT4 glucose transporter (Ginsberg *et al.* 1981, 1982; Elmendorf 2004), mitochondrial respiration (Budin *et al.* 2018), and cold adaptation in poikilotherms are all impaired when cell membranes contain an excess of SFAs (Guschina and Harwood 2006; Svensk *et al.* 2013). Conversely, ω-3 PUFAs are required for the activity of TRP channels that regulate blood pressure (Caires *et al.* 2017), while excess UFA content in phospholipids leads to ferroptotic lipid peroxidation (Yang *et al.* 2016), increased protein aggregation (Fanning *et al.* 2019) and leakiness (Manni *et al.* 2018), which is metabolically very costly (Hulbert and Else 1990).

**Figure 1:**
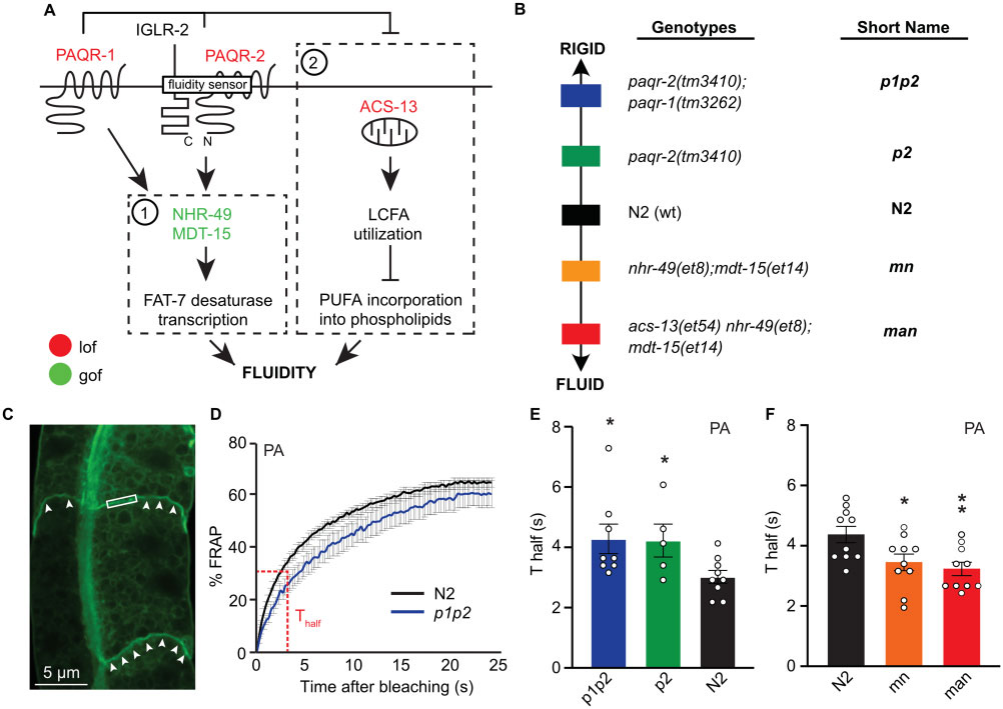
Genetic titration of membrane fluidity measured using FRAP. (A) Model of the molecular pathways leveraged in this study. (B) Overview of the five strains studied, including color coding along the fluidity/rigidity gradient and short names used in most figures. (C) Illustration of a FRAP experiment, showing a *pGLO-1::GFP-CAAX*-positive intestinal membrane. The rectangle indicates the size of the area to be bleached; arrowheads point to well-defined intestinal membranes. (D) Example of a FRAP curve illustrating the measurement of the *T*_half_. (E, F) T_half_ obtained from FRAP experiments for the five strains studied when challenged with palmitic acid-loaded OP50 (PA); the N2 strain was included as reference in both sets of experiments: * and ** indicate *P* < 0.05 and *P* < 0.01 *vs* N2, respectively.

The field of membrane homeostasis has so far lacked a genetically defined animal model spanning the range of membrane composition/fluidity, from excessively rigid/SFA-rich to excessively fluid/UFA-rich. Such models have been presented for bacteria (Budin *et al.* 2018), and can be created in artificial membranes by varying the SFA/PUFA composition (Manni *et al.* 2018; Ballweg *et al.* 2020). Here, we leveraged our previous work on membrane homeostasis regulators in *Caenorhabditis elegans* to study five strains that represent a genetic titration of membrane composition/fluidity (Figure 1A). In particular, our previous work identified the *C. elegans* AdipoR homologs PAQR-1 and PAQR-2 as playing partially redundant roles in maintaining membrane fluidity (Svensk *et al.* 2013, 2016; Busayavalasa *et al.* 2020; Devkota *et al.* 2021), a function that is conserved in the mammalian AdipoRs (Ruiz *et al.* 2019b, 2021). The PAQR-2 protein senses membrane fluidity and is activated when rigidification promotes its interaction with the obligate partner protein IGLR-2: the activated complex promotes FA desaturation and incorporation of UFAs into phospholipids, hence restoring fluidity (Svensk *et al.* 2016; Busayavalasa *et al.* 2020). The membranes of the *paqr-2* mutant therefore contain an excess of SFAs and are rigid, and the *paqr-2* mutant is therefore intolerant of the rigidifying effects of cold or dietary SFAs (Svensk *et al.* 2013; Devkota *et al.* 2017). In addition, this mutant shows a withered morphology of the thin membranous tail tip, decreased brood size, poor locomotion, reduced lifespan, and impaired autophagy at low temperatures (Svensson *et al.* 2011; Svensk *et al.*^2013^, ^2016^; Devkota *et al.* 2017; Chen *et al.* 2019; Lee *et al.* 2019). PAQR-1 appears to constitutively act at a low level to promote the synthesis and incorporation of UFAs into phospholipids, and the *paqr-2* mutant phenotypes are therefore worsened in the *paqr-1 paqr-2* double mutant (Svensson *et al.* 2011; Busayavalasa *et al.* 2020). At the other end of the fluidity spectrum are mutations that promote increased UFA content in phospholipids. These include gain-of-function alleles of the transcriptional regulators NHR-49 (a nuclear hormone receptor) and MDT-15 (a mediator subunit homologous to MED15) that promote desaturase expression (Svensk *et al.* 2013). Loss-of-function mutations in ACS-13 also indirectly promote channeling of PUFAs into membrane phospholipids by decreasing their utilization in mitochondria (Ruiz *et al.* 2019a). Here, we combined mutant alleles of these membrane regulators to generate a group of five strains spanning a wide range of membrane composition and fluidity, and found contrasting effects on several cellular and physiological traits, including permeability, lipid peroxidation, growth at different temperatures, tolerance to SFA-rich diets, lifespan, brood size, oogenesis, vitellogenin trafficking, and autophagy during starvation.

## Materials and methods

### *Caenorhabditis elegans* strains and cultivation

The wild-type *C. elegans* reference strain N2, the transgene carrying strains *RT130* [*pwIs23* (*vit-2::GFP*)], *OD95* (*ItIs37 [(pAA64) pie-1p::mCherry::his-58 + unc-119(+)*] IV; *ItIs38* [*pie-1p::GFP::PH(PLC1delta1*) + *unc-119(+)*]), QC114 [*etEx2* (*glo-1p::GFP::ras-2 CAAX + rol-6(su1006)*)] and the single mutant alleles are available from the *C. elegans* Genetics Center (CGC; USA). *GOT17* [*svIs143* (*nhx-2p::mCherry::lgg-1*)] was a kind gift from Gautam Kao (Raj *et al.* 2019). Unless otherwise stated, experiments were performed at 20°C, using the *Escherichia coli* strain OP50 as food source, which was maintained on LB plates kept at 4°C (restreaked every 6–8 weeks), and single colonies were picked for overnight cultivation at 37°C in LB medium before being used to seed NGM plates (Sulston and Hodgkin 1988). Plates containing glucose or NP40 were prepared by adding the respective stock solutions to cooled NGM after autoclaving.

### Pre-loading of *E. coli* with fatty acids

Stocks of 0.1 M PA (Sigma) dissolved in ethanol or 3.1 M EPA (Sigma) were diluted in LB media to final concentrations of 2 mM, inoculated with OP50 bacteria, then shaken overnight at 37°C. The bacteria were then washed twice with M9 to remove FAs and growth media, diluted to equal OD600, concentrated 10X for PA and 20X for EPA by centrifugation, resuspended in M9, and seeded onto NGM plates lacking peptone (200 ml/plate). Worms were added the following day.

### Growth and other assays

For length measurement studies, synchronized L1s were plated onto test plates seeded with *E. coli*, and worms were mounted and photographed 144 hours (15°C experiments) or 72 hours (all other experiments) later. The length of 20 worms were measured using imageJ (Schneider *et al.* 2012). Other assays have also been previously described in details: total brood size (*n* ≥ 4) (Svensson *et al.* 2011), lifespan (*n* = 100) (Svensson *et al.* 2011), pharyngeal pumping rate (*n* = 10, each monitored for 20 seconds) (Axang *et al.* 2007), locomotion (*n* = 10) (Tajsharghi *et al.* 2005).

### Lipidomics

Lipidomics samples were composed of ∼3000 synchronized L4 larvae grown overnight on OP50-seeded 9 cm plates at 15°C, 20°C, or 25°C. Four plates per genotype or treatment were harvested and lipids extracted in parallel on the same day. Worms were washed three times with M9, pelleted and stored at −80°C until analysis. For lipid extraction, the pellet was sonicated for 10 minutes in methanol and then extracted according to published methods (Lofgren *et al.* 2016). Lipid extracts were evaporated and reconstituted in chloroform: methanol [1:2] with 5 mM ammonium acetate. This solution was infused directly (shotgun approach) into a QTRAP 5500 mass spectrometer (Sciex) equipped with a TriVersa NanoMate (Advion Bioscience) as described previously (Jung *et al.* 2011). Phospholipids were measured using precursor ion scanning (Ekroos *et al.* 2003; Ejsing *et al.* 2009). To generate the phospholipid composition (as mol%) the signals from the individual phospholipids (area under the m/z peak in the spectra) were divided by the signal from all detected phospholipids of the same class. The data were evaluated using the LipidView software (Sciex), and the complete lipid composition data are provided in Supplementary Table S1. The data were further analyzed using Qlucore Omics Explorer n.n (Qlucore AB) for analysis. The data were normalized for the purpose of heat map visualization (mean = 0; variance = 1).

### Fluorescence recovery after photobleaching (FRAP)

FRAP experiments in *C. elegans* were carried out using a membrane-associated prenylated GFP reporter expressed in intestinal cells as described previously (Devkota and Pilon 2018) and using a Zeiss LSM700inv laser scanning confocal microscope with a 40X water immersion objective. Briefly, the GFP positive membranes were photobleached over a rectangular area (25 × 4 -pixels) using 30 iterations of the 488 nm laser with 50% laser power transmission. Images were collected at a 12-bit intensity resolution over 256 × 256 pixels (digital zoom 4X) using a pixel dwell time of 1.58 µs, and were all acquired under identical settings. The recovery of fluorescence was traced for 25 seconds. Fluorescence recovery and *T*_half_ were calculated as described previously (Svensk *et al.* 2016).

### Vitellogenin assay

Bleach synchronized worms carrying the vitellogenin (*vit-2::GFP*) reporter were spotted on NGM plates and incubated at 20°C. After 72 hours of incubation at 20°C, they were washed, mounted on agarose pads, and then observed with a Zeiss Axioscope microscope. Worms were scored based on normal or enhanced enrichment or abnormally localized vitellogenin.

### Germline membrane morphology

Bleach synchronized worms carrying the *ItIs38* [*pie-1p::GFP::PH(PLC1delta1*) + *unc-119(+)*] transgene were spotted on NGM plates and incubated at 20°C. After 48 hours of incubation at 20°C, they were washed and shifted to different temperatures. 24 hours later (48 hours in the case of the *p1p2* and *p2* strains shifted to 15°C), the worms were washed, mounted on agarose pads, and then imaged with a Zeiss Axioscope microscope.

### Germline morphology

Germline morphology was analyzed using Hoechst 34580. Bleach synchronized worms were spotted on NGM plates, incubated at 20°C for 48 hours then transferred to either 15°C, 20°C, or 25°C and incubated for another 24 hours. Worms were then washed and fixed with ice-cold methanol for 5 minutes. The supernatant was then removed and the worms washed twice with PBST. The fixed samples were then stained in 100 ng/ml Hoechst 34580 in PBST for 30 minutes, washed twice with PBST, mounted on agarose pads, and then imaged with a Zeiss Axioscope microscope (Bodhicharla *et al.* 2018).

### Autophagy analysis

Bleach synchronized transgenic worms carrying *lgg-1::mCherry* in the intestine were starved for 6 hours, mounted on agarose pads, imaged with a Zeiss Axioscope microscope and the number of LGG-1::mCherry positive puncta were scored. For experiments with Bafilomycin A1, L1s were incubated in M9 or 25 μM BafA1 for 4 hours before imaging (Aspernig *et al.* 2019). For experiments with temperature, synchronized L1s were grown at 15°C for 16 hours, mounted on agarose pads and then scored for LGG-1::mCherry positive puncta.

### Bodipy-C11 lipid peroxidation assay

Bleach synchronized worms were cultivated at 20°C for 48 hours. For experiments with hydrogen peroxide, worms were washed and incubated with 1 mM hydrogen peroxide for 60 minutes. Worms were then washed and stained with 10 μM BODIPY 581/591 undecanoic acid (BODIPY^581-591^ -C11) solution for 30 minutes and washed three times with M9 buffer. The worms were mounted on agarose slides and imaged using a Zeiss LSM700inv laser scanning confocal microscope. Oxidized and nonoxidized BODIPY^581-591^ -C11 were excited at 488 and 555 and images were collected from emission at 530(30) and 590(30) nm, respectively (Beaudoin-Chabot *et al.* 2019).

### Cell permeability assay

Bleach synchronized worms were cultivated at 20°C for 48 hours. L4 worms were then washed and stained with 100 ng/ml Hoechst 34580 in M9 for 30 minutes, washed twice with M9, mounted on agarose pads and then imaged with a Zeiss Axioscope microscope. Anterior pharyngeal region and posterior tail region were focused in order to avoid the interference from the intestinal gut granules.

### Dietary supplementation assays

Synchronized L1s were plated and grown in plates seeded with OP50 (NGM), PA-loaded OP50 (PA), and EPA-loaded OP50 (EPA). For cell permeability assay, brood size and vitellogenin assay, rigid strains (*p2*, *p1p2*) were initially grown on NGM plates and then supplemented with PA-loaded OP50 since they arrest as early larval stages in the presence of PA. For autophagy analysis, synchronized L1s for all the five strains were grown in different diets for 16 hours, starved for 6 hours, mounted on agarose pads and scored.

### Statistics and replicates

Error bars show the standard error of the mean and t-tests were used to identify significant differences from the wild-type N2 strain, unless otherwise stated. For the lipidomics analysis, ANOVA was used to identify lipids that were significantly different among the groups. Log-rank (Mantel-Cox) test was used to identify significant differences between survival curves. Frequency of cell permeability defect shows the 95% confidence interval determined using *Z-tests*. All experiments, except for the lipidomics analysis, were independently repeated at least twice with similar results, and the statistics shown apply to the presented experimented results. The number of independent biological replicates (*N*) for the experiments performed with the specific conditions described are as follows: FRAP, *N* = 2; lipidomics at different temperatures, *N* = 1; cell permeability assays, *N* = 3; lipid peroxidation assays, *N* = 2; growth at different temperatures/treatments, *N* = 2; brood size, *N* = 3; lifespan, *N* = 3; vitellogenin localization assays, *N* = 2; germline morphology scoring, *N* = 2; autophagy assays (with or without Bafilomycin A1), *N* = 3; dietary supplementation, *N* = 2. Asterisks are used in the figures to indicate various degrees of significance, as follows: *P < 0.05, **P < 0.01, and ***P < 0.001.

### Data availability

The authors affirm that all data necessary for confirming the conclusions of this article are represented fully within the article and its figures. In addition, supplemental figures and the complete lipidomics data are provided as supplementary material available at figshare: https://doi.org/10.25386/genetics.14604807.

## Results

### FRAP confirms the successful genetic titration of membrane fluidity

We studied strains with the *paqr-1(tm3262); paqr-2(tm3410), paqr-2(tm3410)*, N2 (wild-type), *mdt-15(et14); nhr-49(et8)*, and *mdt-15(et14); nhr-49(et8); acs-13(et54)* genotypes, abbreviated *p1p2*, *p2*, N2, *mn* and *man*, respectively (Figure 1B). We knew from previous work that the *p1p2* strain had worse phenotypes (life span, brood size, and so on) than the single *p2* mutant (Svensson *et al.* 2011). We also knew from preliminary experiments that double and triple mutants were required to obtain a growth defect phenotype at 25°C or in the presence of PUFAs. Therefore, we devised a set of mutants with a gradient of gross phenotypic defects that, based on preliminary studies, were likely due to a gradient of fluidity of their membranes. This led to the selection of the five strains shown in Figure 1B. Based on our previous studies of these alleles, we expected these strains to display a graded degree of membrane fluidity in the presence of membrane-rigidifying PA, with the *p1p2* and *p2* strains having the most rigid and the *mn* and *man* strain the most fluid membranes. This was indeed the case, as determined using the fluorescence recovery after photobleaching (FRAP) method to monitor *in vivo* the rate at which membrane-bound prenylated GFP protein can laterally diffuse to repopulate a laser-bleached plasma membrane area in an intestinal cell (Figure 1, C–F). Note that while there is good reproducibility within experiments performed on the same day, there is some variation between experiments performed on separate days, which explains the differences between the N2 worms in Figure 1, E and F. Such variation is likely due to intangibles that affect day-to-day measurements, including variation in the temperature of the room (microscope heat/air conditioning, fan, and so on) which can have a significant effect on membrane fluidity, as well as humidity, exact composition of the dietary *E. coli* and other factors. Note also that no differences in FRAP were detected on NGM plates (Supplementary Figure S1, A and B), either because this standard culture condition does not challenge membrane homeostasis or because any difference is beyond the sensitivity of the FRAP method.

### Genetic titration of membrane composition mirrors temperature adaptation

**Figure 2:**
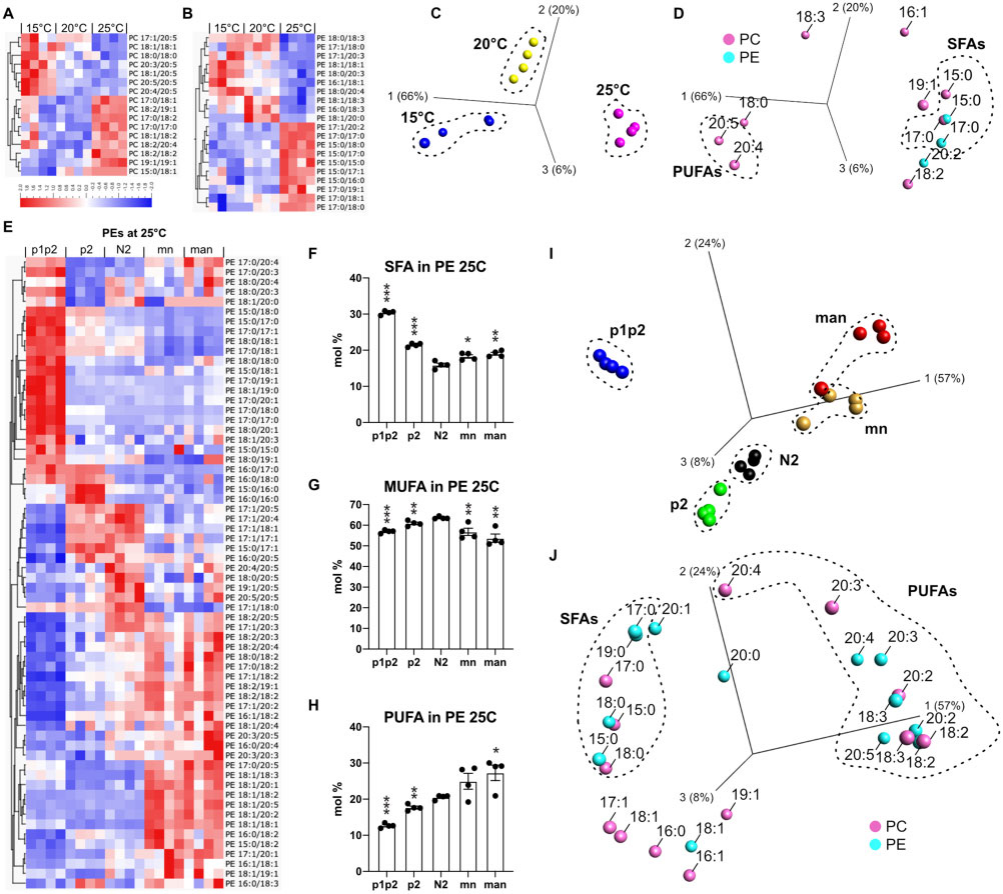
Genetic titration of membrane composition correlates with fluidity. (A, B) Heat maps showing the PC and PE composition in N2 worms grown at 15°C, 20°C, or 25°C. (C, D) Principal component analysis of the PC and PE FA composition clearly separates the three temperatures; only FAs that differed significantly (*q* < 0.05 in ANOVA) are shown. (E) Heat map showing the PE composition of all five strains grown at 25°C. (F, H) SFA, MUFA, and PUFA composition among the PEs of all five strains grown at 25°C. (I, J) Principal component analysis of the PC and PE FA composition clearly separates the five strains grown at 25°C. Only FAs that differed significantly (*q* < 0.05 in ANOVA) are shown in the heat maps and principal component analysis (PCA). *P < 0.05, **P < 0.01, and ***P < 0.001 *vs* N2 in F and G. For the heat maps, the mean for each lipid type was adjusted to 0 and the variance normalized to 1; the scale in A applies also to B and E and reflects standard deviations from the mean. For each condition (genotype or temperature), four plates of worms were grown and processed in parallel. Each of the four columns presented in the heat map for each genotype or temperature represents one of the four plates of the experiment.

*C. elegans*, being a poikilotherm, adapts its membrane composition in response to changes in ambient temperature: the membranes are poorer in SFAs/richer in UFAs at low temperature (*e.g.*, 15°C), with opposite changes at higher temperature (*e.g.*, 25°C) (Tanaka *et al.* 1996). We were able to confirm these adaptive composition changes by cultivating the N2 strain at 15°C, 20°C, and 25°C for 24 hours prior to harvesting and lipidomics analysis. In particular, we found many PCs to carry two PUFA chains at 15°C, while more PCs and phosphatidylethanolamines (PEs) carried only SFAs or monounsaturated fatty acids (MUFAs) at 25°C (Figure 2, A and B). Principal component analysis is an unbiased statistical method to identify variables that most distinguish different sets of samples from each other (Ringner 2008). We found that the changes in FA composition allowed for a clear separation of the samples in a principal component analysis (Figure 2C), and revealed that the main lipid composition differences that occur when N2 worms are cultivated at different temperatures relate to changes in PUFA levels (higher at 15°C, lower at 25°C) and SFA levels (lower at 15°C and higher at 25°C) (Figure 2D).

We then performed an identical analysis on the five strains that genetically titrate membrane fluidity. Figure 2, E–H shows the lipid composition differences among PEs, the most abundant phospholipids in *C. elegans* (Satouchi *et al.* 1993), of worms cultivated at 25°C where the separation among the five strains was clearest as per the principal component analysis. In agreement with the FRAP results, we found that the *p1p2* strain was excessively rich in SFAs while the *man* strain was excessively rich in PUFAs, with the *p2*, N2 and *mn* strains containing intermediate levels of these FAs (Figure 2, E–H). Principle component analysis based on FA composition of PCs and PEs showed that most of the differences among the five strains are explained by excess SFAs among the *p1p2* and *p2* strains, and excess PUFAs among the *mn* and *man* strains. Similar results were obtained also at 15°C and 20°C; the complete lipidomics data for PCs and other temperatures are presented in Supplementary Figures S1 and S2 and Supplementary Table S1.

Based on the FRAP and lipidomics results, we conclude that the five strains studied here do represent an effective titration of membrane fluidity and composition, with *p1p2* and *p2* being most rigid/SFA-rich and *mn* and *man* being the most fluid/PUFA-rich. We then studied these five strains to determine the importance of membrane homeostasis on cellular traits, namely permeability and lipid peroxidation levels, and several physiological traits, namely growth at different temperatures, tolerance to SFA-rich diets, lifespan, brood size, vitellogenin trafficking, oogenesis, and autophagy during starvation.

### Both excess rigidity and excess fluidity lead to membrane leakiness

**Figure 3:**
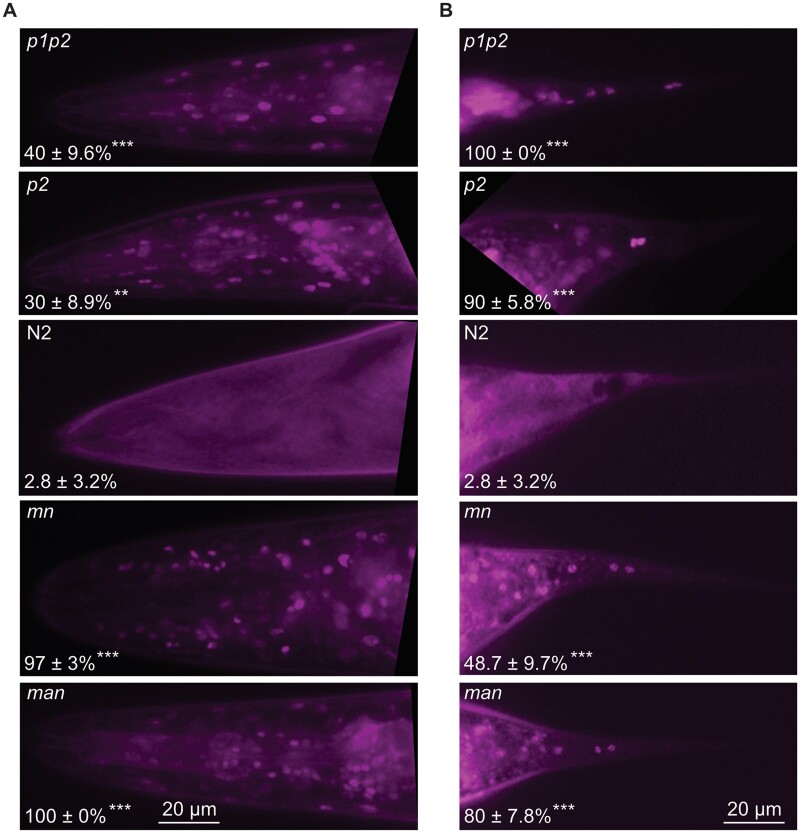
Increased and decreased membrane fluidity both cause membrane leakiness. (A) Head and (B) tails of worms from the different genotypes stained with Hoechst 34580 in M9 for 30 minutes. The percentages of worms with clearly stained nuclei is indicated for each genotype *(p1p2*, *n* = 25; *p2*, *n* = 50; N2, *n* = 35; *mn*, *n* = 39; *man*, *n* = 25) **P < 0.01 and ***P < 0.001 *vs* N2.

Fine-tuning of lipid composition is critical to prevent membrane leakiness. It is generally accepted that increasing UFA levels among phospholipids tends to increase leakiness. What is perhaps less appreciated is that excess SFAs can cause phase separation within the membranes that can lead to loss of membrane integrity and leakage (Leekumjorn *et al.* 2009). Conversely, asymmetric partitioning of PUFAs between the two phospholipid leaflets can actually improve membrane impermeability, especially in situations of high membrane curvature (Manni *et al.* 2018). We used the Hoechst 34580 dye to assess plasma membrane leakiness among the five strains and found that only N2 was impermeable: the dye efficiently crossed the plasma membrane and labeled numerous nuclei in the head and tail (Figure 3, A and B) and throughout the body (Supplementary Figure S3) of *p1p2, p2*, *mn*, and *man* mutant worms cultivated under normal conditions. This is a startling demonstration that membrane homeostasis is critical for the maintenance of membrane integrity, and that deviation toward either excess SFA or UFA content leads to membrane leakiness even under nonstress conditions.

### Lipid peroxidation levels correlate with membrane fluidity/PUFA content

**Figure 4:**
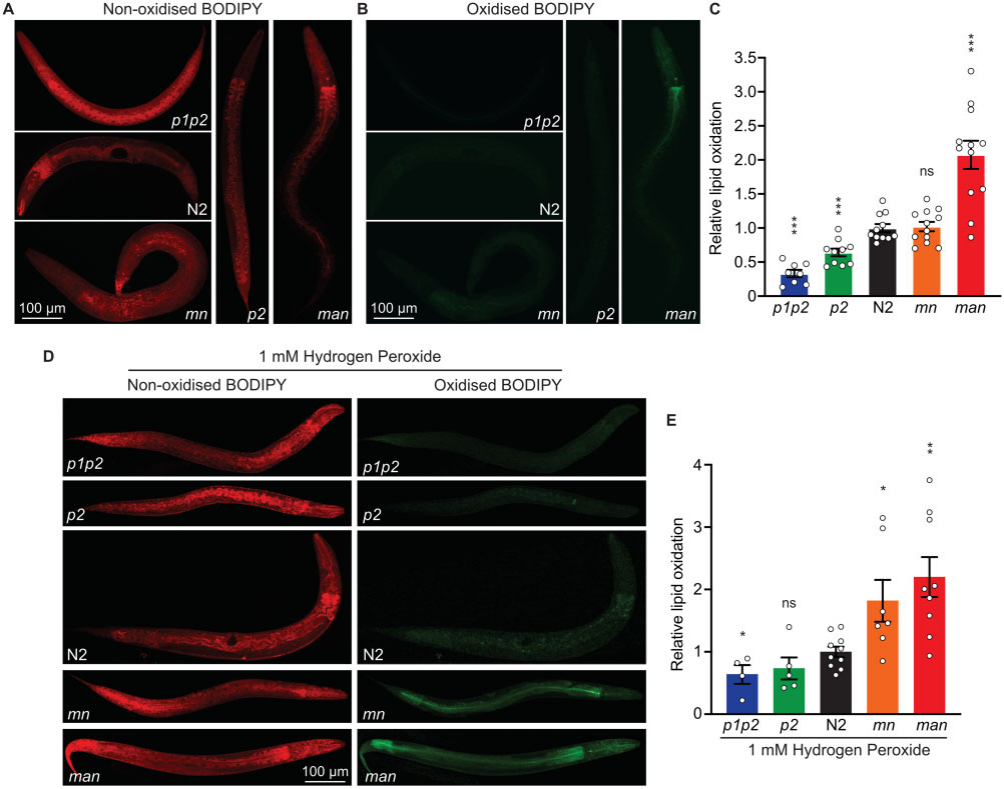
The amount of lipid peroxidation correlates with membrane fluidity. (A) Oxidized and (B) nonoxidized BODIPY^581-591^ -C11 were excited at 488 and 555 and images were collected from emission at 530(30) and 590(30) nm, respectively. (C) Quantification of the relative lipid oxidation; *n* = 12. (D) Oxidized and nonoxidized BODIPY^581-591^ -C11 in five strains treated with 1 mM hydrogen peroxide. (E) Quantification of the relative lipid oxidation treated with hydrogen peroxide; (*n* ≥ 4). *P < 0.05, **P < 0.01, ***P < 0.001 and not significant (ns) *vs* N2.

It is generally accepted that increased PUFA content increases the risk of lipid peroxidation, though this has rarely been systematically studied. Here, we used a BODIPY-C11 reporter assay (Beaudoin-Chabot *et al.* 2019) to compare the degree of lipid peroxidation in the five *C. elegans* strains and found a clear correlation between lipid peroxidation levels and PUFA content. Specifically, the *p1p2* strain showed the lowest, and the *man* strain the highest levels of lipid peroxidation, with *p2*, N2 and *mn* falling in between and in that order (Figure 4, A–C). These measurements were performed without specific addition of an oxidative stressor and therefore reflect the endogenous levels of lipid peroxidation among the five strains. We also found that the addition of 1 mM hydrogen peroxide to the worm suspension resulted in a marked increase in oxidized BODIPY-C11 in all five strains, which validates this lipid peroxidation assay (Figure 4, D and E). We conclude that the levels of lipid peroxidation do indeed correlate with PUFA levels.

### Excess membrane fluidity or rigidity impacts growth in different temperatures or diets

**Figure 5:**
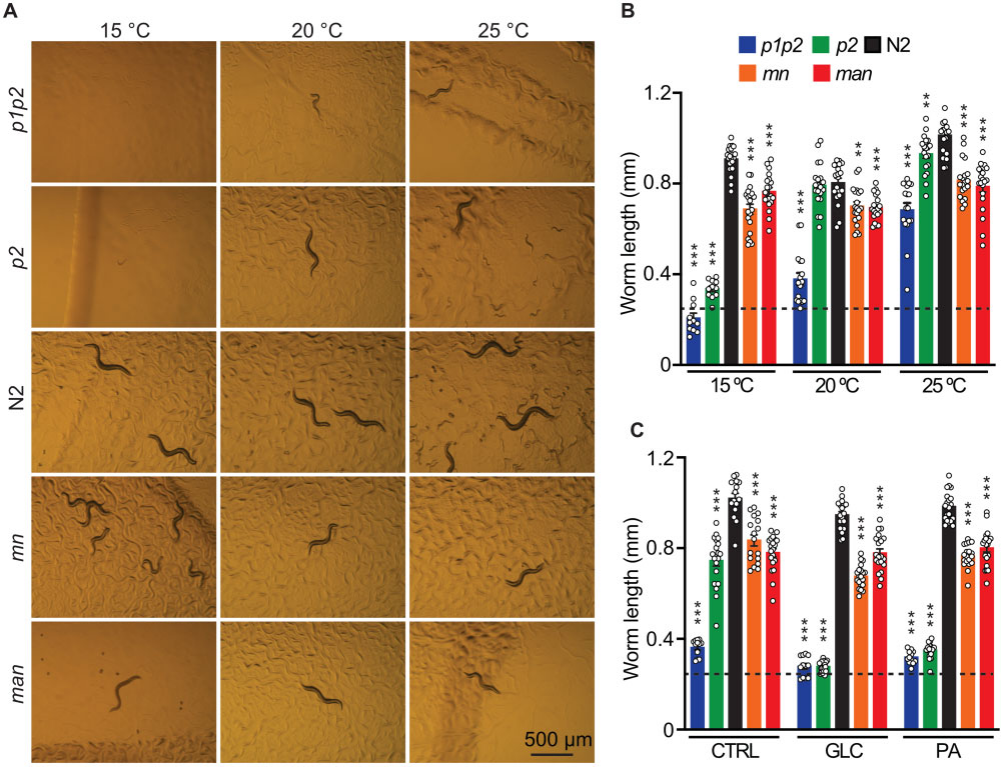
A genetic series titrating sensitivity to membrane rigidification challenges. (A) Photographs of worms with the various genotypes studied were grown at three different temperatures for 72 hours (20°C and 25°C) or 144 hours (15°C). (B) Quantification of lengths from A. (C) Quantification of lengths of worms grown for 72 hours on control plates (CTRL), glucose plates (GLC), or on a diet of 2 mM PA-loaded OP50 (PA). The dashed line represents the average length of L1 worms at the start of the experiment. **P < 0.01, and ***P < 0.001 *vs* N2.

The ability to grow, *i.e.*, length reached within a given period of time in different culture conditions, was also compared among the five strains studied. We found that the *p1p2* and *p2* strains grew poorly or not at all at 15°C or in the presence of either glucose [which is readily converted to SFAs by the dietary *E. coli* (Devkota *et al.* 2017)] or palmitate (a 16:0 SFA; Figure 5, A–C). These strains grew better at 20°C and much better at 25°C (Figure 5, A and B), suggesting that increased temperature partially compensates for the excess SFA content in the phospholipids of these strains. The *mn* and *man* grew relatively well under all conditions tested, though never as well as the N2 wild-type strain and actually grew shorter than the *paqr-2* mutant at 25°C. This suggests that excess PUFA content/fluidity is always detrimental but may not be as harmful as excessive rigidification, except at higher temperature. All five strains grew well on a diet of *E. coli* pre-loaded with 2 mM eicosapentaenoic acid (EPA; C20:5) both at 20°C and 25°C (Supplementary Figure S4, A and B), but we noted that the addition of 0.05% NP40, which acts as a fluidizing agent (Svensk *et al.* 2016), caused a reduction in the growth of the *mn* and *man* strain relative to the growth of the *p2* and N2 strains (Supplementary Figure S4C), suggesting that these strains are particularly intolerant to fluidizing conditions. We conclude that growth of excessively SFA-rich/rigid strains is impaired by low temperature or SFA-rich diets, while the growth of excessively UFA-rich/fluid strains is impaired at higher temperatures or by fluidizing agents.

### Excess membrane fluidity or rigidity are both deleterious to reproduction and lifespan

**Figure 6:**
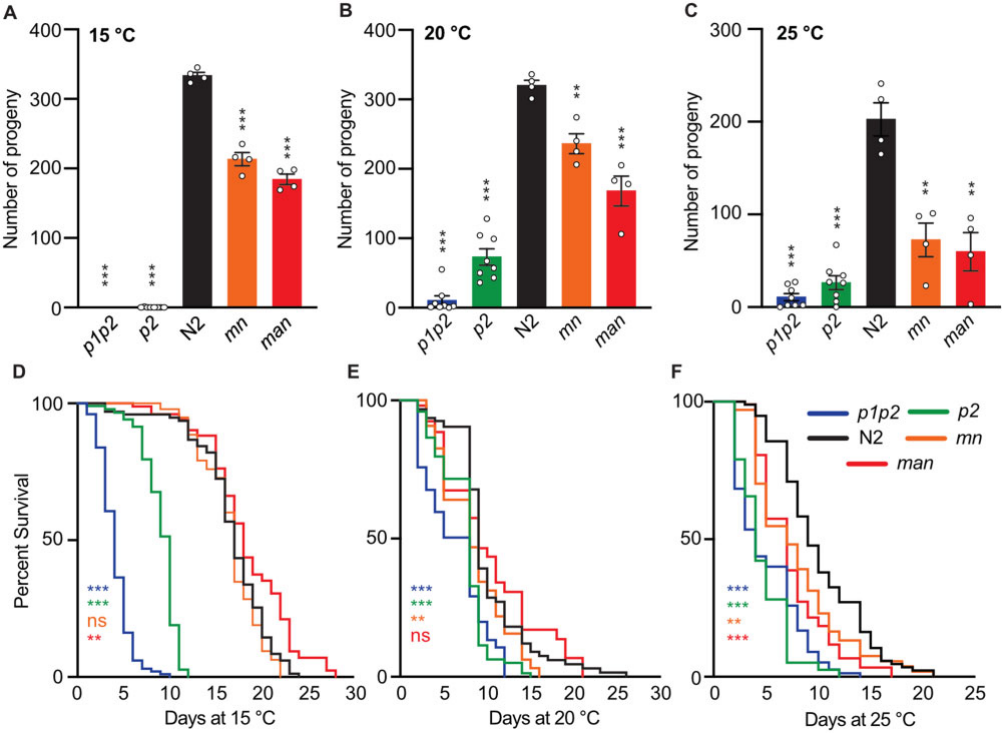
Impaired membrane homeostasis decreases brood sizes and lifespans at three temperatures. (A–C) Brood sizes (*n* ≥ 4) and (D–F) lifespans (*n* = 100) were determined for all five strains studied. **P < 0.01, ***P < 0.001 and not significant (ns) *vs* N2.

To evaluate the importance of membrane homeostasis for the fitness of *C. elegans*, we determined the average brood size and lifespan at 15°C, 20°C, and 25°C for the five strains spanning a gradient of membrane composition/fluidity. Consistent with previous studies, the *p1p2* and *p2* strains failed to reproduce and had dramatically reduced lifespans at 15°C, produced small broods but showed improved life spans at 20°C and 25°C (Figure 6, A–F). The *p1p2* and *p2* strains also moved poorly and had a slight reduction in pharyngeal pumping rates at 20°C (Supplementary Figure S4, D and E). Together, these results demonstrate the essentiality of maintaining membrane fluidity at lower temperatures, but also that preventing excess SFA content is important at all temperatures. Conversely, the *mn* and *man* strains also showed reduced brood sizes at all temperatures, but especially at 25°C, and also showed a reduction in lifespan at 25°C (Figure 6, A–F). These strains had no locomotion or pharyngeal pumping defects (Supplementary Figure S4, A and B). These results demonstrate the importance of preventing excess PUFA content/fluidity at all temperatures but especially at 25°C, and also suggest that excessive membrane fluidity may not be as harmful as excessive rigidification, or that truly harmful excess fluidity is genetically difficult to achieve. Importantly, the pronounced fragility of the *p1p2* and *p2* strains at 15°C and the fragility of the *mn* and *man* strains at 25°C suggest that these strains are not simply “sick” but rather exhibit more severe phenotypes at temperatures that exacerbate the genetically induced membrane composition defects.

### Contrasting effects of excess rigidity/fluidity on oogenesis

**Figure 7:**
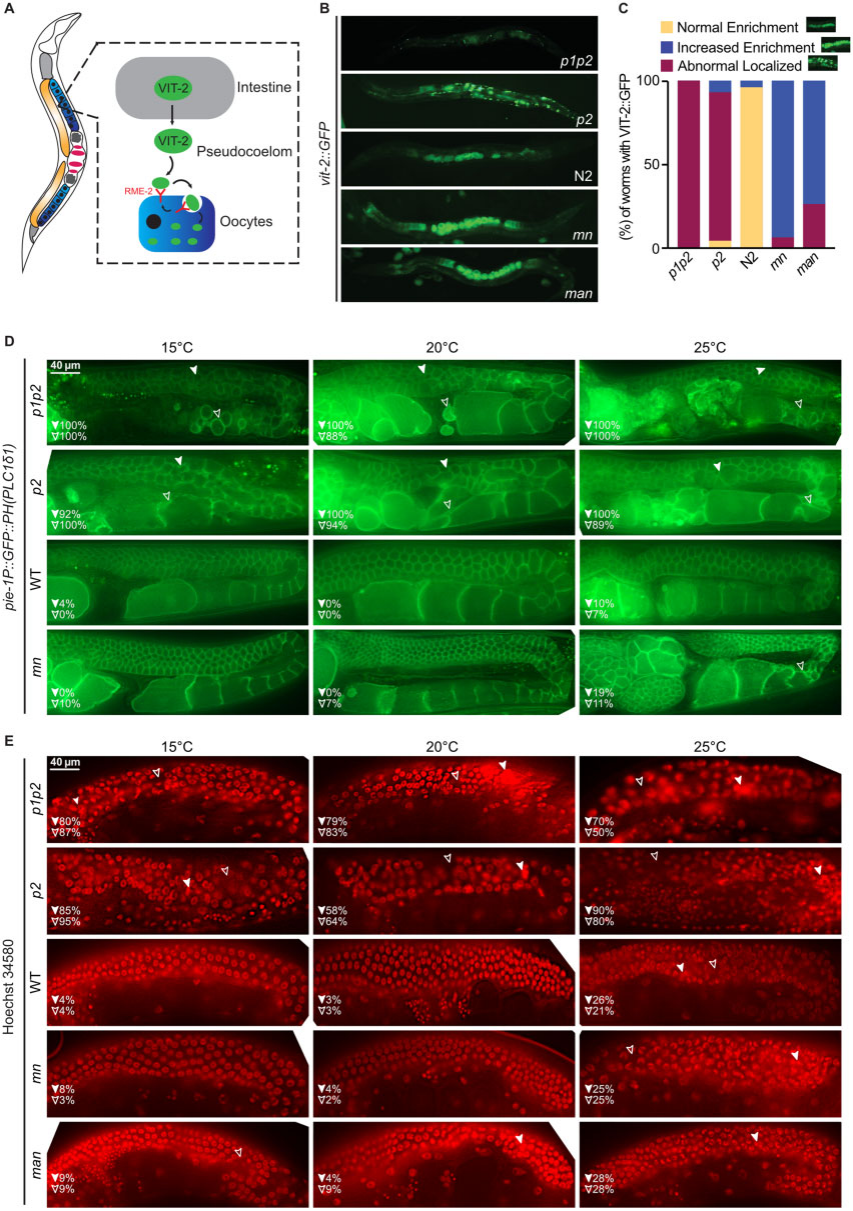
The vitellogenin mislocalization and oogenesis phenotypes correlate with membrane rigidity. (A) Illustration of VIT-2 trafficking from the intestine to oocytes via RME-2-dependent endocytosis. (B) Representative images of VIT-2::GFP in the five strains. (C) Quantification of the VIT-2 GFP phenotypes classified into three categories (*n* = 50). (D) Representative images of the *pie-1P::GFP::PH(PLCδ1)* reporter labeling the membranes within the gonad arms in four strains and at three different temperatures. The filled arrowheads represent the frequency of worms with abnormal early germline; the empty arrowheads represent the frequency of worms with defective oocytes. (E) Representative images of Hoechst stained gonads in the five strains and at three different temperatures. The filled arrowheads represent the frequency of worms with nuclear aggregates in the germline; the empty arrowheads represent the frequency of worms with abnormal gaps in the germline.

Strains with either excess membrane rigidity (*p1p2* and *p2*) or fluidity (*mn* and *man*) had severely reduced brood sizes (Figure 6A). To better understand these brood size defects, we used a VIT-2::GFP reporter to visualize the trafficking of vitellogenins, lipoproteins that ferry lipids and other nutrients from the intestine to the gonad where they normally accumulate within developing oocytes (Figure 7A). We observed remarkably contrasting phenotypes between the excessively rigid and fluid strains. The *p1p2* strain showed only low levels of VIT-2::GFP while the *p2* strain exhibited a stronger expression; both strains ectopically accumulated the VIT-2::GFP reporter in the pseudocoelomic cavity rather than into the developing oocytes (Figure 7, B and C). In contrast, the *mn* and *man* strains showed much more intense VIT-2::GFP accumulation within oocytes compared to the wild-type N2 strain, suggesting excessive import of vitellogenin (Figure 7, B and C). Thus, excess rigidification and excess fluidity cause opposite vitellogenin accumulation defects. We then used a *pie-1P::GFP::PH(PLCδ1)* reporter to visualize the membranes within the gonads of the five strains. In the wild-type N2, this reporter presents a regular honeycomb pattern in the distal portion of the gonad arm, including in the mitotic/meiotic transition zone, and reveals the plasma membranes of increasingly mature oocytes in the more proximal region as well as in early cleavage stage embryos in the uterus (Figure 7D). In contrast, the *p1p2* and *p2* strains nearly always (>95% of gonad arms) show disorganized mitotic/meiotic transition zones, gaps within the gonad arms, and disorganized oocytes and embryos at 15°C, 20°C, and 25°C (Figure 7D). The *mn* strain had relatively normal honeycomb patterns in their distal ends but showed many instances of abnormal oocytes as well as an excess accumulation of late cleavage-stage embryos in the uterus (Figure 7D), especially at 25°C where ∼20% of animals contained hatchlings. Hoechst staining to visualize nuclei within the gonad arms confirms severe disorganization within the proximal regions *in p1p2* and *p2* mutants, especially at 15°C (Figure 7E); few or no specific defects were observed in the *mn* and *man* strains using Hoechst, though all strains showed some nuclei disorganization at 25°C (Figure 7E). We conclude that vitellogenin trafficking is impaired in opposite ways by excessively rigid or fluid membranes, and that early events of oogenesis are more affected in the *p1p2* and *p2* strain while later events are more affected in the *mn* and *man* strains. These gonad defects likely contribute to the reduced brood sizes in the mutant strains.

### Contrasting effects of excess rigidity/fluidity on starvation-induced autophagy

**Figure 8:**
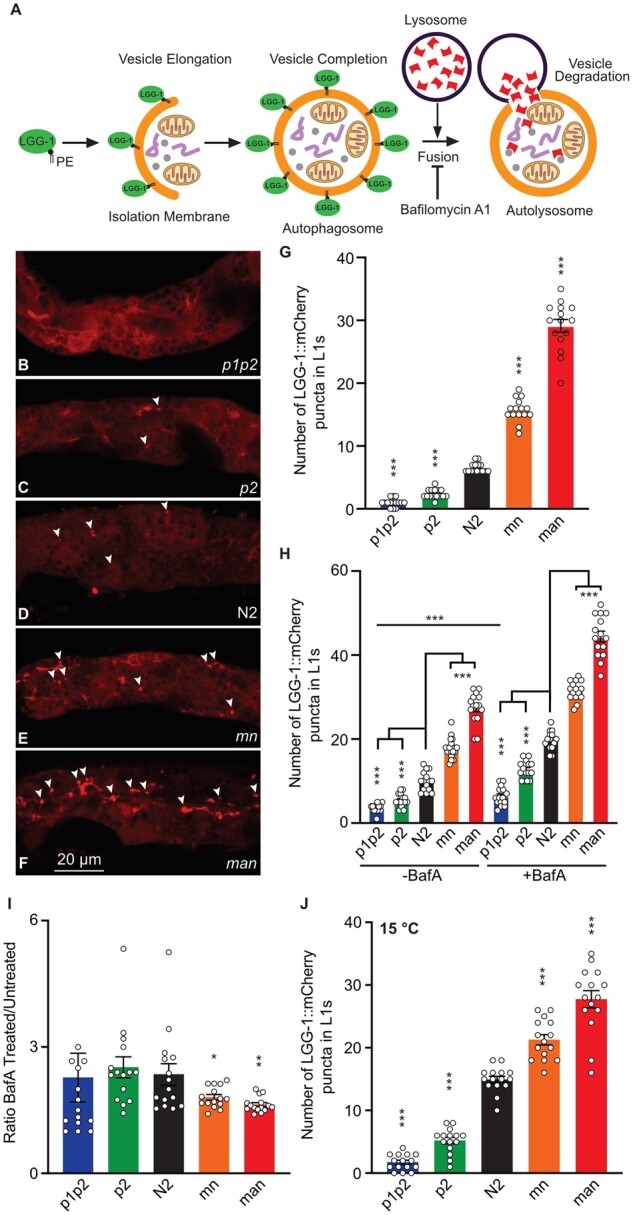
Autophagy in response to starvation correlates with membrane fluidity. (A) Overview of the autophagy process with emphasis on the LGG-1 protein used here as a reporter of autophagy. (B–F) Representative images of LGG-1::mCherry in five *C. elegans* strains starved for 6 hours; arrowheads indicate puncti scored as autophagosomes. (G) Quantification of the LGG1::mCherry puncti in worms from the five different strains; *n* = 15. (H) Quantification of the LGG1::mCherry puncti in worms from the five different strains incubated as L1s in M9 buffer or M9 buffer containing 25 μM BafA1 for 4 hours before imaging; *n* = 15. (I) Ratio of BafA1 treated/untreated. (J) Quantification of the LGG1::mCherry puncti in worms from the five different strains at 15°C; *n* = 15. *P < 0.05, **P < 0.01, ***P < 0.001 and not significant (ns) *vs* N2.

We previously showed that eating-defective mutants upregulate autophagy within the intestine (Morck and Pilon 2006). Here, we used a LGG-1::mCherry reporter to monitor the presence of autophagosomes in overnight-starved L1s of the five different strains studied here (Figure 8A). Remarkably, there was a perfect correlation between fluidity/PUFA content and the number of LGG-1::mCherry puncti in starved L1s, with the *p1p2* strain containing almost no autophagosomes and, at the other extreme, the *man* strain containing over 30 such puncti, which is double the number found in the wild-type N2 worms (Figure 8, B–G). We then used bafilomycin to inhibit the fusion of autophagosomes with lysosomes (Zhang *et al.* 2015), and found that the number of LGG-1::mCherry puncti then increased in all strains (Figure 8H), though slightly less so in the *mn* and *man* strains as determined by measuring the ratio of bafilomycin-treated over untreated samples (Figure 8I). This suggests that *mn* and *man* animals have increased autophagosome formation combined with impaired degradation in the autophagolysosome or impaired resolution of the autophagolysosome. In addition, shifting the worms from 20°C to 15°C is another way to induce autophagy (Chen *et al.* 2019) which, again, revealed a perfect correlation between fluidity/PUFA content and the number of LGG-1::mCherry puncti (Figure 8J). We conclude that it is mostly the rate of autophagosome formation that is impaired in rigid strains and increased in fluid strains in response to either starvation or shift to 15°C, and that the excess fluidity also affects autophagosome processing.

### Dietary lipid supplementation modulates several phenotypes

**Figure 9:**
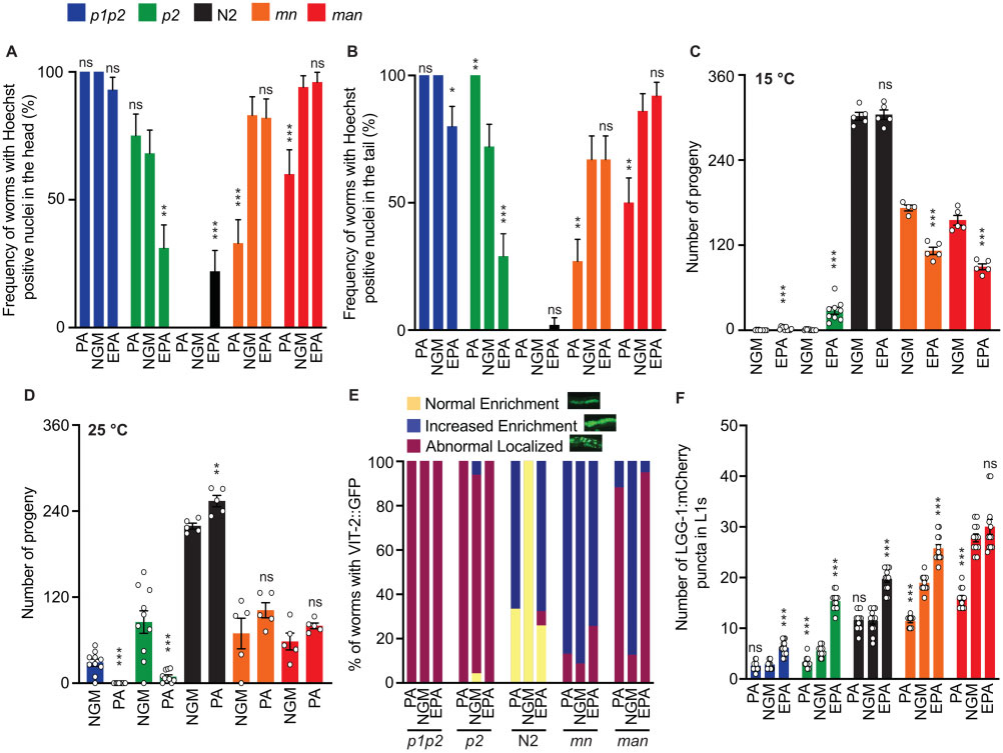
Dietary lipid supplementation ameliorates cellular defects observed among the five strains. (A) Frequency of worms with Hoechst positive nuclei in the head, grown on normal plates (NGM) and when supplemented with palmitic acid-loaded OP50 (PA) and eicosapentaenoic acid-loaded OP50 (EPA); (*n* ≥ 10). (B) Frequency of worms with Hoechst positive nuclei in the tail, grown on normal plates (NGM) and when supplemented with palmitic acid-loaded OP50 (PA) and eicosapentaenoic acid-loaded OP50 (EPA); (*n* ≥ 10). (C) Brood sizes (*n* ≥ 4) for all the five strains at 15°C grown on normal plates (NGM) and when supplemented with eicosapentaenoic acid-loaded OP50 (EPA). (D) Brood sizes (*n* ≥ 4) for all the five strains at 25°C grown on normal plates (NGM) and when supplemented with palmitic acid-loaded OP50 (PA). (E) Quantification of the VIT-2::GFP phenotypes classified into three categories when grown on normal plates (NGM) and when supplemented with palmitic acid-loaded OP50 (PA) and eicosapentaenoic acid-loaded OP50 (EPA); (*n* = 50). (F) Quantification of the LGG1::mCherry puncti in worms from the five different strains grown at 20°C on normal plates (NGM) and when supplemented with palmitic acid-loaded OP50 (PA) and eicosapentaenoic acid-loaded OP50 (EPA); (*n* = 12). *P < 0.05, **P < 0.01, ***P < 0.001 and not significant (ns) *vs* NGM for each genotype.

Dietary intervention can alter the composition of cellular membranes, and probably more so in mutant strains lacking genes important for membrane homeostasis. We, therefore, tested whether including PA (an SFA) or EPA (a PUFA) would worsen or suppress some of the phenotypes that were identified in the present study (Figure 9). Among the more interesting results, we found that PA significantly suppressed the following phenotypes in the *mn* and/or *man* strains: membrane permeability (Figure 9, A and B) and the excess autophagy phenotype (Figure 9F). PA also showed a nonsignificant tendency to improve the brood sizes of these strains at 25°C (Figure 9D) and caused abnormal localization of VIT-2::GFP in the *man* strain (Figure 9E). Conversely, EPA partially but significantly suppressed several phenotypes in the *p1p2* and/or *p2* strains including: membrane permeability (Figure 9, A and B), brood size at 15°C (Figure 9C) and the autophagy defect (Figure 9F). The fact that PA suppressed *mn* and *man* phenotypes while EPA suppressed *p1p2* and *p2* phenotypes suggests that dietary supplements can ameliorate the membrane composition defects in these strains and, therefore, implies that most of the observed defects are due to FA imbalance in the membranes and not pleiotropic effects. Note that the VIT-2 loading defects may be consequent, and not causal, to the sterility since dietary supplementation of EPA to *p1p2* and *p2* partially rescues the brood size phenotype but not the VIT-2 loading defect.

### Reproducibility of the experiments

Except for the lipidomics analysis, all experiments were performed multiple times and yielded generally reproducible results; all data presented in the form of graphs or figure panels came from one representative experiment. Supplementary Figure S5 shows an example of experimental replicates for several key experiments: permeability assay using the dye DAPI rather than Hoechst 34580 (Supplementary Figure S5A; this is not strictly an experimental replicate but shows that the permeability defect in mutant strains is not specific to one dye), lipid peroxidation assay (Supplementary Figure S5B), brood sizes and lifespans at 15°C, 20°C, and 25°C (Supplementary Figure S5, C–H), locomotion assay (Supplementary Figure S5), pharyngeal pumping rate (Supplementary Figure S5J), VIT-2::GFP localization (Supplementary Figure S5K) and autophagy assay with and without bafilomycin (Supplementary Figure S5L).

## Discussion

We have established a genetic series in *C. elegans* that titrates membrane fluidity. The range of fluidity among the five strains correlates well with membrane composition and sensitivity to membrane rigidifying challenges such as changes in temperature or SFA content in the diet. Importantly, we also show that several cellular processes are affected in a graded manner along the fluidity axis and that the phenotypes are, at least partially, rescued by dietary supplements anticipated to correct the membrane fluidity defects (*i.e.*, SFAs rescued the *mn* and *man* strains while UFAs rescued the *p1p2* and *p2* strains). This study demonstrates the critical roles of membrane homeostasis in maintaining cell and organismal physiology, not only to membrane-specific challenges but also for vital processes such as cold adaptation, growth, oogenesis, prevention of oxidative damage, autophagy, and preserving the impermeable quality of cellular membranes. Indeed, given that every cellular process either takes place at the level of a membrane or within a membrane-bound compartment, it is hardly surprising that most, if not all, cellular processes are dependent on successful membrane homeostasis.

**Figure 10:**
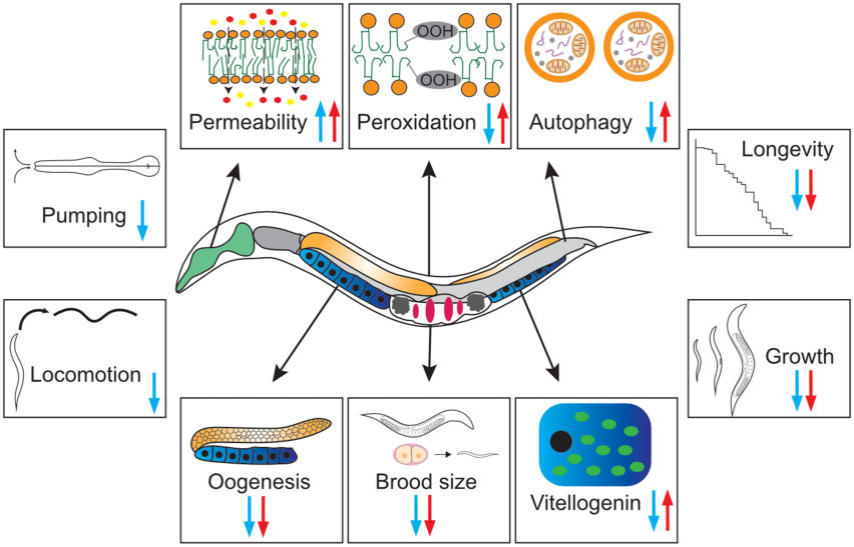
Membrane homeostasis defects impair multiple processes. Blue and red arrows indicate the direction of change for a given process in strains with excess rigidity (*p1p2* and *p2*) or fluidity (*mn* and *man*), respectively.

As summarized in Figure 10, excess SFA content/membrane rigidity (indicated by blue arrows), as in the *p1p2* and *p2* strains, showed multiple striking phenotypes. These strains were nonviable at 15°C or when fed an SFA-rich diet, had poor locomotion, dramatically reduced brood size and lifespan, accumulated vitellogenins in the pseudocoelom rather than in oocytes, had defective early oogenesis, impaired formation of autophagosomes during starvation and were leaky to the dye Hoechst 34580. These strains also showed significantly lower levels of lipid peroxidation.

It is relatively easy to find mechanistic explanations connecting excess membrane SFA content/rigidity with these phenotypes. For example, vitellogenin-dependent transport of yolk is a membrane-dependent process at several levels: yolk is secreted by the intestine and is within minutes loaded into oocytes by receptor-mediated endocytosis (Grant and Hirsh 1999; Hall *et al.* 1999; Kuo *et al.* 2013) after which yolk appears in a membrane-bound compartment, *i.e.*, the yolk granule (Hall *et al.* 1999). Yolk transport to the oocyte depends on correct FA composition and PUFA-deficient mutants accumulate an excess of yolk material in the pseudocoelom, as we observed here with the SFA-rich strains *p1p2* and *p2* (Chen *et al.* 2016). With respect to the VIT-2::GFP phenotypes, the germ cell production defects in the *p2* mutant may be sufficient to explain the accumulation of VIT-2 in the coelom if its production from the intestine then exceeds uptake by the defective germline. Conversely, increased uptake of VIT-2 in *mn* and *man* may indicate an increase in VIT-2 expression or secretion by the intestine, which is sufficient to cause excess yolk accumulation in oocytes (Balklava *et al.* 2016). Autophagy is also an eminently membrane-driven process (Orsi *et al.* 2010). During autophagy, phagophores (isolation membranes) form throughout the cytosol, expand and enwrap cytosol and organelles, generating the double membrane autophagosomes in a process that depends on the transfer of ER lipids to nascent phagophores (Maeda *et al.* 2019). The autophagy process is sensitive to both membrane composition, packing and curvature. For example, the lipid kinase VPS34 that orchestrates autophagy is activated by UFAs and negative membrane curvature (Ohashi *et al.* 2020), while lipidation of the LC3/GABARAP family of autophagy protein depends on the membrane-curvature-sensing domain of the Atg3 protein (Nath *et al.* 2014). Speculatively, poor locomotion in the SFA-rich/rigid membrane strains may be explained by reduced synaptic transmission, which depends on PUFA-rich synaptic vesicles (Takamori *et al.* 2006; Antonny *et al.* 2015) or, by analogy with aging cardiac muscles, because of ER stress caused by the excess lipid saturation (Lee *et al.* 2020). As mentioned earlier, excess SFAs can also lead to membrane leakiness because they lead to domains with high order phases that lack plasticity and could compromise membrane integrity under high curvature (Leekumjorn *et al.* 2009; Manni *et al.* 2018). In *C. elegans*, mutations in the desaturases *fat-2* and *fat-6; fat-7* result in the disruption of the permeability barrier in embryos and, consequently, increased embryonic lethality (Watts *et al.* 2018), which is consistent with our present findings.

Excess PUFA content/membrane fluidity, as in the *mn* and *man* strains also showed several distinct phenotypes (indicated by red arrows in Figure 10). In particular, these strains had decreased brood sizes at all temperatures, showed reduced lifespan at 25°C, excess accumulation of vitellogenin in oocytes accompanied by accumulation of abnormal embryos within the uterus, increased starvation-induced autophagy and lipid peroxidation, and had leaky membranes. For some of these traits, the directions of change in the *mn* and *man* strains were opposite to that of the *p1p2* and *p2* strains and may have a similar mechanistic basis. In particular, yolk import into oocytes and autophagy may be more efficient in a UFA-rich membrane environment, and increased lipid peroxidation and membrane permeability are expected and known consequences of increased UFA content. In particular, enrichment of ω-6 PUFAs occurs during the starvation response in *C. elegans* and promotes the increased autophagy essential for lifespan extension (O'Rourke *et al.* 2013)*.* On the other hand, membrane phospholipids containing an excess of PUFAs are highly susceptible to peroxidation triggered by free radicals, thereby leading to the generation of reactive phospholipid hydroperoxides that can trigger ferroptosis (Yang *et al.* 2016; Conrad *et al.* 2018) and, in *C. elegans,* affects germ-cell morphology (Perez *et al.* 2020) and shorten lifespan (Sakamoto *et al.* 2020). Similarly, increasing PUFA content in model membranes leads to increased disorder associated with leakiness and even flip-flopping of phospholipids (Olbrich *et al.* 2000; Armstrong *et al.* 2003), which is consistent with the dye-permeability observed in the *mn* and *man* strains.

Membrane properties, here simplified merely as “fluidity,” but which in reality include lateral mobility, packing, curvature, thickness, compressibility, charge and other properties, clearly impact on a multiplicity of cellular processes (Mouritsen 2011; Radanovic *et al.* 2018). The importance of preserving near-optimal properties is made clear from the present studies, but is also supported by numerous other studies. For example, deep-sea fish adapt to the rigidifying effects of high pressure by increasing their membrane UFA content (Cossins and Macdonald 1989), just as poikilotherms increase UFA content to compensate for the rigidifying effects of low temperature (Cossins and Macdonald 1989; Guschina and Harwood 2006). The precise FA composition appears to be less important than maintaining the biophysical properties of membranes in a variety of organisms. For example, yeast adapt to changes in the relative abundance of PCs and PEs in membranes, which influences membrane fluidity (Dawaliby *et al.* 2016), by changing the lengths and saturation of the FAs in their phospholipids (Boumann *et al.* 2006). Also, mammals adapt to excess exogenous long-chain PUFAs by increasing both SFA and cholesterol levels (Levental *et al.* 2020).

From an energetics point of view, the leakiness of the membranes observed in both the excessively rigid and excessively fluid strains appear particularly costly. Even in healthy cells, it has been estimated that well over 50% of the cellular energy goes toward maintenance of gradients, including Na^+^ gradients, Ca^2+^ gradients and maintaining mitochondrial membrane potential against proton leaks (Buttgereit and Brand 1995; Rolfe and Brown 1997; Hulbert and Else 2000). The heavy metabolic cost that must be incurred by increased membrane leakiness in the *p1p2*, *p2*, *mn*, and *man* strains surely contributes to their decreased growth, brood size, and general fitness.

In conclusion, we leveraged our understanding of membrane homeostasis in *C. elegans* to create a panel of five strains that represent a genetic titration of SFA/PUFA content and membrane fluidity, which we verified using FRAP and lipidomics. These strains exhibit strong phenotypes in nearly all cellular or physiological assays tested, with the excessively rigid strains often being defective in an opposite direction to that of the excessively fluid strains. It is likely that most of the observed phenotypes are secondary to the primary membrane defects in the mutant strains. However, as a word of caution, we note that the mutant alleles used in the present study likely have other functions besides maintenance of membrane homeostasis, which obviously complicates the interpretation of the results. This is particular true of ACS-13, NHR-49 and MDT-15, which are implicated in a wide range of functions that include regulating PUFA utilization and FA beta oxidation in mitochondria as well as regulating responses to various stressors (Taubert *et al.* 2006; Goh *et al.* 2014; Pukkila-Worley *et al.* 2014; Moreno-Arriola *et al.* 2016; Hu *et al.* 2018; Ruiz *et al.* 2019a; Shomer *et al.* 2019). However, even keeping this caveat in mind, it is hoped that the strain panel introduced here will prove useful to study the cellular and physiological consequences of impaired membrane homeostasis which, as we have shown here, are many.
